# Supplementary material for: Cell invasion during competitive growth of polycrystalline solidification patterns
Source: Nat Commun. 2023 Apr 19;14:2244. doi: 10.1038/s41467-023-37458-0 (PMC10115863; doi:10.1038/s41467-023-37458-0)
Supplement: Supplementary file 1 — Supplementary Information [file 41467_2023_37458_MOESM1_ESM.pdf]

Cell invasion during competitive growth of polycrystalline solidification patterns

Supplementary Information

Supplementary Methods

Experimental setup. In the Bridgman experimental setup, a linear temperature profile, with a homogeneous temperature gradient of an amplitude G in the direction x, is established by controlling a low temperature furnace and a higher temperature furnace on both sides of a thermally insulated (adiabatic) zone. At rest, a planar solid-liquid interface forms close to the alloy liquidus temperature, i.e. 330.85 K for a succinonitrile (SCN)-0.24wt% camphor alloy. When the sample crucible is pulled towards the colder area, the interface initially moves towards the colder area because its growth is slower than the pulling velocity V, which is also the target steady state growth velocity. During this initial interface recoil, the concentration at the interface on the liquid side increases due to the rejection of camphor by the growing solid of lower camphor solubility than the liquid phase. The resulting increase of the solute gradient at the interface leads to an increase of the interface velocity. Once the interface velocity exceeds a critical value that depends on G and on the alloy phase diagram, the planar interface becomes morphologically unstable to perturbations [1–3]. Then, small ridges and poxes appear along the interface (see e.g. Fig. 2 of Ref. [3] and early stage of Supplementary Movie 1), which ultimately develop into grain boundaries (GBs) and cells, respectively [3–5]. The pattern coarsens as cells compete with one another through thermal and solute fields, until enough cells have been eliminated and a stable array spacing is established among surviving cells. It is worth noting that GBs here are all low-angle GBs, which may also be referred to as subboundaries [6–8].

Experimental parameters. Various (G, V) conditions were investigated within the DECLIC-DSI. In previous studies [9–11], we focused on a narrow range of parameters that resulted in oscillating dynamics of cellular arrays, for instance for $V = 0.5$ to 1.5 μm/s at $G = 19$ K/cm. In the

current study, we focus on experiments in which we observed at least one converging GB, that is for $V = 1.5$ and 2.0 µm/s at G = 19 K/cm. Material parameters for a SCN-0.24wt% camphor alloy are listed in Supplementary Table 1 and have been discussed elsewhere [10,12,13].

Experimental observations. As shown in Supplementary Movie 1 for $V = 1.5$ µm/s and Supplementary Fig. 1 below for $V = 2.0$ µm/s, the growing microstructure consists of several cellular grains with different crystal orientations, which can easily be distinguished due to their distinct drifting directions, i.e. their overall growth direction projected in the y-z projection plane (illustrated with arrows in Supplementary Fig. 1). Thus, GBs (red lines in Supplementary Fig. 1) form between grains. When the two different grains grow towards each other, the GB is called converging, and cells directly adjacent to the GB progressively eliminate one another by impingement [14–19]. These successive elimination events give rise to the GB roughening and its complex morphologies (e.g. Supplementary Fig. 1). Supplementary Movies 2 and 3 show a SC emerging at $V = 1.5$ µm/s. We also observe emergence of a SC for $V = 2.0$ µm/s and $G = 19$ K/cm (Supplementary Movie 4 and Supplementary Fig. 2). Whenever a SC emerges, it drifts within its host grain up to several hours following its own crystal orientation (Supplementary Fig. 2b-d). This continuous SC growth leads to GB branching and tubular defects (Figs. 2c and 3c in the Letter).

Post-processing of experimental results. Shortly after the pulling of the sample starts, the planar solid-liquid interface destabilizes (see early stage of Supplementary Movie 1), and the primary spacing λ in cellular arrays increases due to the growth competition among cells [3,11]. An initial transient oscillatory growth regime follows [9–11]. We discard this transient regime in our estimation of crystalline orientations by performing our analysis exclusively on well-developed microstructures, growing as an overall solidification front at a steady undercooling and velocity, i.e. after $t \approx 3.5$ h for $V = 1.5$ µm/s or $t \approx 1.4$ h for $V = 2.0$ µm/s. We use image processing software *Visilog* and *ImageJ* to extract the center locations in the y-z plane for every cell, as well as their evolution in time [10,11]. We then extract the drifting velocity for each cell, V_d, which allows us to: (1) group them into different grains, and (2) estimate the three-dimensional crystalline orientation of each grain. Given that the observed microstructures are growing at a steady velocity V along

the x-direction, as imposed by the pulling velocity of the crucible, we can extract the main growth direction angle θ_d with respect to the temperature gradient (and pulling) direction x with the simple geometrical relation

$$\tan\theta_d = V_d/V. \tag{1}$$

The growth direction θ_d usually differs from the crystal orientation θ [16,18,20–22]. The relation between a growth angle θ_d and a crystal orientation angle θ is discussed later in this document, in light of established theory and additional thorough phase-field validation. In Fig. 2 of the Letter with $V = 1.5$ µm/s, we measured $V_d = 0.09$ µm/s and $\theta_d = 3.4°$ for the left grain, and $V_d = 0.04$ µm/s and $\theta_d = 1.5°$ for the right grain. In Fig. 1d-h in the Letter, the lower-left grain had $V_d = 0.07$ µm/s and $\theta_d = 2.5°$, and other grains were similar as in Fig. 2 of the Letter.

Simulation model. Here we use a PF model that derives from a thin-interface asymptotic analysis, such that it reduces to the corresponding sharp-interface problem while representing the solid-liquid interface as a diffuse interface with a width that is much larger than the actual physical thickness of the interface [23,24]. The model neglects solid-state diffusion of solute because it is much slower in the solid than in the liquid phase. Hence, accounting for a predominantly diffusive transport regime in microgravity conditions, solute transport occurs through pure Fickian diffusion in the liquid phase. The model includes an anti-trapping term that corrects the spurious solute trapping due to the width of the diffuse interface [25,26]. The model is quantitative within a dilute binary alloy limit, such that the phase diagram of the solid-liquid interface equilibrium can be described using constants for the interface solute partition coefficient k and the alloy liquidus slope m. The resulting model, briefly described below, is essentially similar to that described in Refs. [26,27], only with a few minor additions to improve its computational efficiency [10,16,19].

Since the transport of heat is much faster than the transport of solute, we consider the temperature field as an externally imposed boundary condition. We assume that the temperature profile is frozen in the setup with a fixed temperature gradient G along the x-axis, which results in the common assumption referred to as frozen temperature approximation [9–11,16,19,22,27,28]. The validity and limitations of this approximation, specifically in the context of the DECLIC-DSI experiments,

are discussed in Ref. [3]. In the frame of the material, moving at a velocity V with respect to the furnace, the temperature T at a position along the x axis is thus given by

$$T = T_0 + G(x - Vt), \tag{2}$$

where T_0 at $x = 0$ is a reference temperature, here chosen to be the solidus temperature of the alloy. Within this temperature field, the evolution of the preconditioned phase field ψ and of the normalized solute concentration U with time t are described by [16,26,27]

$$\left[1-(1-k)\frac{\tilde{x}-\widetilde{V}\tilde{t}}{\tilde{l}_T}\right] a_s(\mathbf{n})^2\frac{\partial\psi}{\partial t} = \vec{\nabla}\left[a_s(\mathbf{n})^2\right]\vec{\nabla}\psi + a_s(\mathbf{n})^2\left[\nabla^2\psi - \varphi\sqrt{2}|\vec{\nabla}\psi|^2\right]$$
$$+\sum_{q=x,y,z}\left[\partial_q\left(|\vec{\nabla}\psi|^2 a_s(\mathbf{n})\frac{\partial a_s(\mathbf{n})}{\partial(\partial_q\psi)}\right)\right] + \sqrt{2}\left[\varphi - \lambda_c(1-\varphi^2)\left(U + \frac{\tilde{x}-\widetilde{V}\tilde{t}}{\tilde{l}_T}\right)\right], \tag{3}$$

$$\Big(1+k-(1-k)\varphi\Big)\frac{\partial U}{\partial t} = \widetilde{D}\,\vec{\nabla}\cdot\left[(1-\varphi)\vec{\nabla}U\right] + \vec{\nabla}\cdot\left[\Big(1+(1-k)U\Big)\frac{(1-\varphi^2)}{2}\frac{\partial\psi}{\partial t}\frac{\vec{\nabla}\psi}{|\vec{\nabla}\psi|}\right]$$
$$+\left[1+(1-k)U\right]\frac{(1-\varphi^2)}{\sqrt{2}}\frac{\partial\psi}{\partial t}, \tag{4}$$

where

$$U \equiv \frac{1}{1-k}\left[\frac{2\,c/c_l^0}{(1+k)-(1-k)\varphi} - 1\right], \tag{5}$$

$c_l^0 = c_\infty/k$ is the equilibrium concentration on the liquid side of the interface at the reference temperature T_0 for a nominal alloy composition c_∞ with $0 < k < 1$, and φ is the standard phase-field variable with $\varphi = -1$ $(+1)$ for the liquid (solid) before preconditioning, i.e. $\varphi = \tanh(\psi/\sqrt{2})$. Space and time are scaled with respect to the diffuse interface width W and the relaxation time τ_0 at T_0, respectively [26]. Thus, the dimensionless diffusivity $\widetilde{D}$ is given by

$$\widetilde{D} = \frac{D\tau_0}{W^2} = a_1 a_2 \frac{W}{d_0} \tag{6}$$

with $a_1 = 5\sqrt{2}/8$ and $a_2 = 47/75$ from thin-interface asymptotics [23,24]. The interface solute capillary length d_0 and the coupling factor λ_c are

$$d_0 = \frac{\Gamma}{|m|c_\infty(1/k-1)}, \tag{7}$$

and

$$\lambda_c = a_1 \frac{W}{d_0}, \tag{8}$$

where Γ is the Gibbs-Thomson coefficient of the solid-liquid interface. The dimensionless thermal length is

$$\tilde{l}_T = \frac{l_T}{W} = \frac{l_T}{d_0} \frac{1}{W/d_0}, \tag{9}$$

with $\tilde{x} \equiv x/W$ and $\widetilde{V} \equiv V\tau_0/W$. The term $a_s(\mathbf{n})$ in Eq. (3) represents the anisotropy of the surface energy, which is discussed in details later in this document (subsection of Crystal orientation in the numerical model). Finally, in order to incorporate the effect of thermal fluctuations, we add noise by introducing a random perturbation onto the preconditioned phase field evolution equation [9,10,19,22], using a uniform random distribution with a given amplitude F_ψ.

Numerical implementation. Eqs. (3) and (4) are discretized spatially with centered finite differences with a homogenous grid spacing Δx, and using an explicit Euler time stepping scheme with a constant time step Δt [16,19,26–28]. For stability of the explicit scheme, we use a time step

$$\Delta t = R_S \frac{(\Delta x)^2}{6\widetilde{D}}, \tag{10}$$

where $0 < R_S < 1$ is a constant safety factor, here set to 0.9. Compared to the model as presented in earlier articles [26,27], we use additional methods to enhance computational efficiency. First, the PF model uses a preconditioned phase field ψ, instead of using the classical phase field φ, through the nonlinear change of variable $\varphi \equiv \tanh(\psi/\sqrt{2})$. This change of variable makes the calculations stable for coarser numerical grids [29]. Second, we use the CUDA programing platform [30] to perform simulations in parallel on Nvidia™ GPU (Graphics Processing Unit) architectures. For multi-GPU calculations, we use direct peer-to-peer communication between GPUs [31,32]. For the simulation of large domains (e.g. simulation in Fig. 3 in the Letter), we use up to eight GPUs simultaneously. Within the explicit Euler time stepping scheme, the random perturbation on the ψ field is introduced as

$$\psi^{t+\Delta t} = \psi^t + \Delta t \partial_t \psi + F_\psi \sqrt{\Delta t} \zeta, \tag{11}$$

where F_ψ is a noise strength, and ζ is a random number, generated at each grid point and each time step with a uniform distribution, within the $[-0.5, +0.5]$ range.

Crystal orientation in the numerical model. *Solid-liquid interface anisotropy.* We use a standard form of the surface tension anisotropy with cubic symmetry given by [33]

$$a_s(\mathbf{n}) = (1 - 3\epsilon_4)\left[1 + \frac{4\varepsilon_4}{1-3\varepsilon_4}\left(n_x^4 + n_y^4 + n_z^4\right)\right], \tag{12}$$

where ε_4 is a measure of the anisotropy strength, and $\mathbf{n} = (n_x, n_y, n_z)$ is the unit vector normal to the interface pointing towards the liquid, where n_x, n_y, and n_z are components of $\mathbf{n}$ along the x, y, and z axes, respectively. To impose the three-dimensional (3D) orientation of a grain in the PF model, we use three orientation angles α, γ, and β, following three successive steps: (1) rotate about the z-axis by an angle α (Supplementary Fig. 3a), (2) rotate about the y'-axis by an angle γ (Supplementary Fig. 3b), and (3) rotate about the x''-axis by an angle β (Supplementary Fig. 3c). Resulting crystal axes (x''', y''', z''') are thus rotated by angles (α, β, γ) with respect to the fixed Cartesian axes (x, y, z) as schematically illustrated in Supplementary Fig. 3. The x''' direction represents the main growth direction of a grain. This x''' axis is used to identify the preferred growth direction using spherical angles θ and ϕ, as explained later.

Implementation of the rotated anisotropy. The rotation with angles (α, γ, β) can be written in matrix forms as

$$\begin{pmatrix} x' \\ y' \\ z' \end{pmatrix} = R_1 \begin{pmatrix} x \\ y \\ z \end{pmatrix} = \begin{pmatrix} \cos\alpha & \sin\alpha & 0 \\ -\sin\alpha & \cos\alpha & 0 \\ 0 & 0 & 1 \end{pmatrix} \begin{pmatrix} x \\ y \\ z \end{pmatrix}, \tag{13}$$

$$\begin{pmatrix} x'' \\ y'' \\ z'' \end{pmatrix} = R_2 \begin{pmatrix} x' \\ y' \\ z' \end{pmatrix} = \begin{pmatrix} \cos\gamma & 0 & \sin\gamma \\ 0 & 1 & 0 \\ -\sin\gamma & 0 & \cos\gamma \end{pmatrix} \begin{pmatrix} x' \\ y' \\ z' \end{pmatrix}, \tag{14}$$

and

$$\begin{pmatrix} x''' \\ y''' \\ z''' \end{pmatrix} = R_3 \begin{pmatrix} x'' \\ y'' \\ z'' \end{pmatrix} = \begin{pmatrix} 1 & 0 & 0 \\ 0 & \cos\beta & \sin\beta \\ 0 & -\sin\beta & \cos\beta \end{pmatrix} \begin{pmatrix} x'' \\ y'' \\ z'' \end{pmatrix}. \tag{15}$$

The crystal axes (x''', y''', z''') thus derive from the fixed Cartesian axes (x, y, z) through

$$\begin{pmatrix} x''' \\ y''' \\ z''' \end{pmatrix} = \mathbf{R} \begin{pmatrix} x \\ y \\ z \end{pmatrix}, \tag{16}$$

where the full rotation matrix is

$$\mathbf{R} = R_3 R_2 R_1 = \begin{pmatrix} r_{11} & r_{12} & r_{13} \\ r_{21} & r_{22} & r_{23} \\ r_{31} & r_{32} & r_{33} \end{pmatrix} \tag{17}$$

with

$$\begin{aligned}
r_{11} &= \cos\alpha \cos\gamma, \\
r_{12} &= \sin\alpha \cos\gamma, \\
r_{13} &= \sin\gamma, \\
r_{21} &= -\sin\alpha \cos\beta - \cos\alpha \sin\beta \sin\gamma, \\
r_{22} &= \cos\alpha \cos\beta - \sin\alpha \sin\beta \sin\gamma, \\
r_{23} &= \sin\beta \cos\gamma, \\
r_{31} &= \sin\alpha \sin\beta - \cos\alpha \cos\beta \sin\gamma, \\
r_{32} &= -\cos\alpha \sin\beta - \sin\alpha \cos\beta \sin\gamma, \\
r_{33} &= \cos\beta \cos\gamma.
\end{aligned}$$

Inversely, the fixed Cartesian coordinates (x, y, z) can be retrieved by

$$\begin{pmatrix} x \\ y \\ z \end{pmatrix} = \mathbf{R}^{-1} \begin{pmatrix} x''' \\ y''' \\ z''' \end{pmatrix} = \begin{pmatrix} r_{11} & r_{21} & r_{31} \\ r_{12} & r_{22} & r_{32} \\ r_{13} & r_{23} & r_{33} \end{pmatrix} \begin{pmatrix} x''' \\ y''' \\ z''' \end{pmatrix}. \tag{18}$$

Thus, in Eq. (3), the first derivatives of ψ with respect to crystal axes (x''', y''', z''') are

$$\partial_{x'''}\psi = \partial_{x'''}x\partial_x\psi + \partial_{x'''}y\partial_y\psi + \partial_{x'''}z\partial_z\psi, \tag{19}$$

$$\partial_{y'''}\psi = \partial_{y'''}x\partial_x\psi + \partial_{y'''}y\partial_y\psi + \partial_{y'''}z\partial_z\psi, \tag{20}$$

$$\partial_{z'''}\psi = \partial_{z'''}x\partial_x\psi + \partial_{z'''}y\partial_y\psi + \partial_{z'''}z\partial_z\psi, \tag{21}$$

or in a matrix form

$$\begin{pmatrix} \partial_{x'''} \\ \partial_{y'''} \\ \partial_{z'''} \end{pmatrix} \psi = \begin{pmatrix} r_{11} & r_{12} & r_{13} \\ r_{21} & r_{22} & r_{23} \\ r_{31} & r_{32} & r_{33} \end{pmatrix} \begin{pmatrix} \partial_x \\ \partial_y \\ \partial_z \end{pmatrix} \psi. \tag{22}$$

The second order derivative with respect to x''' can be calculated as

$$\partial_{x'''x'''}\psi = r_{11}\partial_x(\partial_{x'''}\psi) + r_{12}\partial_y(\partial_{x'''}\psi) + r_{13}\partial_z(\partial_{x'''}\psi). \tag{23}$$

Similarly, developed expressions of second derivatives are

$$\begin{aligned}
\partial_{x'''x'''}\psi &= r_{11}^2\partial_{xx}\psi + r_{12}^2\partial_{yy}\psi + r_{13}^2\partial_{zz}\psi + 2(r_{11}r_{12}\partial_{xy}\psi + r_{12}r_{13}\partial_{yz}\psi + r_{11}r_{13}\partial_{zx}\psi), \\
\partial_{y'''y'''}\psi &= r_{21}^2\partial_{xx}\psi + r_{22}^2\partial_{yy}\psi + r_{23}^2\partial_{zz}\psi + 2(r_{21}r_{22}\partial_{xy}\psi + r_{22}r_{23}\partial_{yz}\psi + r_{21}r_{23}\partial_{zx}\psi), \\
\partial_{z'''z'''}\psi &= r_{31}^2\partial_{xx}\psi + r_{32}^2\partial_{yy}\psi + r_{33}^2\partial_{zz}\psi + 2(r_{31}r_{32}\partial_{xy}\psi + r_{32}r_{33}\partial_{yz}\psi + r_{31}r_{33}\partial_{zx}\psi), \\
\partial_{x'''y'''}\psi &= r_{11}(r_{21}\partial_{xx}\psi + r_{22}\partial_{xy}\psi + r_{23}\partial_{xz}\psi) + r_{12}(r_{21}\partial_{yx}\psi + r_{22}\partial_{yy}\psi + r_{23}\partial_{yz}\psi) \\
&\quad + r_{13}(r_{21}\partial_{zx}\psi + r_{22}\partial_{zy}\psi + r_{23}\partial_{zz}\psi), \\
\partial_{y'''z'''}\psi &= r_{21}(r_{31}\partial_{xx}\psi + r_{32}\partial_{xy}\psi + r_{33}\partial_{xz}\psi) + r_{22}(r_{31}\partial_{yx}\psi + r_{32}\partial_{yy}\psi + r_{33}\partial_{yz}\psi) \\
&\quad + r_{23}(r_{31}\partial_{zx}\psi + r_{32}\partial_{zy}\psi + r_{33}\partial_{zz}\psi), \\
\partial_{z'''x'''}\psi &= r_{31}(r_{11}\partial_{xx}\psi + r_{12}\partial_{xy}\psi + r_{13}\partial_{xz}\psi) + r_{32}(r_{11}\partial_{yx}\psi + r_{12}\partial_{yy}\psi + r_{13}\partial_{yz}\psi) \\
&\quad + r_{33}(r_{11}\partial_{zx}\psi + r_{12}\partial_{zy}\psi + r_{13}\partial_{zz}\psi).
\end{aligned}$$

Here we only studied configurations with $\beta = 0°$ while exploring α and γ orientation angles, such that coefficients of the rotation matrix may be simplified to $r_{11} = \cos\alpha\cos\gamma$, $r_{12} = \sin\alpha\cos\gamma$, $r_{13} = \sin\gamma$, $r_{21} = -\sin\alpha$, $r_{22} = \cos\alpha$, $r_{23} = 0$, $r_{31} = -\cos\alpha\sin\gamma$, $r_{32} = -\sin\alpha\sin\gamma$, and $r_{33} = \cos\gamma$.

Bi-crystalline implementation. We distinguish the two grains in the simulations by introducing an integer grain index field $p(x, y, z, t)$ [16,19]. In the liquid far from the solid-liquid interface, the index value is initialized as $p_{i,j,k} = 0$ at a location of discrete spatial coordinates (i, j, k) along the x, y, and z axes, respectively. This value is updated as soon as $(1 - \varphi^2_{i,j,k}) \geq 0.001$, i.e. when the solid-liquid interface approaches. The new index value is estimated by using the summation of p indices across the 27 neighbors

$$S_{i,j,k} = \mathrm{sign}\left\{\sum_{i-1}^{i+1}\sum_{j-1}^{j+1}\sum_{k-1}^{k+1} p_{(i,j,k)}\right\}, \tag{24}$$

such that the index is then fixed to $p = -1$ in one grain and $p = +1$ in the other grain. The current approach thus does not account for triple junctions and grain boundary motion in the solid state, which would anyway be inexistent due to the assumption of no solid-state diffusion.

Conversion to experimentally measured spherical angles. When α and γ have non-zero values, the main crystal orientation axis, i.e. the x''' axis (red arrow in Fig. 4 in the Letter), differs from the x axis of the temperature gradient. For convenience, we convert angles (α, γ) at $\beta = 0°$ introduced in previous subsections into spherical angles (θ, ϕ), directly extracted from the experimental measurements, and used throughout the discussion in the Letter. From the components (r_{11}, r_{12}, r_{13}) of Eq. (17), one can readily calculate spherical angles (θ, ϕ) using standard geometrical relationships, such as $\cos\theta = r_{11}$ and $\tan\phi = (r_{13}/r_{12})$. Supplementary Table 2 summarizes (α, γ) and calculated (θ, ϕ) angles used in PF simulations. In single-grain simulations, ϕ is the (counterclockwise) angle with respect to the $y+$ direction. In bi-crystalline simulations, the orientation ϕ_1 of grain 1 (represented in blue) is measured with respect to the $y+$ direction (as in single-grain simulations), while the orientation ϕ_2 of grain 2 (represented in red) is measured from the $y-$ direction, both of them measured counterclockwise, as illustrated in Fig. 4 in the Letter. Note that we always set $\beta = 0°$, which does not have a strong influence in the cellular regime under investigation here, but would matter in the dendritic regime when individual dendrites exhibit non-axisymmetrical features such as fins and sidebranches [33].

Simulation parameters. We use material parameters for a SCN-0.24wt% camphor alloy identified and discussed in previous studies [10–13,34]. These parameters are listed in Supplementary Table 1. PF simulations exclusively focus on one set of experimental parameters, namely with $V = 1.5$ µm/s and $G = 19$ K/cm. The corresponding numerical parameters are also listed in Supplementary Table 1. Simulations use a random noise strength $F_\psi = 0.01$, except for simulations in Supplementary Fig. 5 that are performed with $F_\psi = 0$.

Simulation configurations. *Drift dynamics of a single grain.* The relationship between the crystal orientation of a grain θ and its actual growth direction θ_d was initially derived from confined thin sample experiments [20,21] and afterwards validated both 2D and confined 3D phase-field simulations [16,18,22,35]. Here, we further validated that this relationship still stands for different 3D patterns. To do so, we generated microstructure arrays growing at steady state at $V = 1.5$ µm/s and $G = 19$ K/cm with different pattern symmetry, namely hexagonal (Supplementary Fig. 4a) and FCC-like (faced centered cubic, Supplementary Fig. 4b), as well as a thin-sample confined-3D geometry (Supplementary Fig. 4c). Using the resulting arrays of imposed primary spacing λ generated with a well-oriented grain with $\theta = \phi = 0°$ as initial conditions, we then restarted the simulations with different (θ, ϕ) orientations in order to assess the dependence of θ_d upon θ, ϕ, and λ. The procedure is, to a large extent similar to that presented in previous papers [10]. First, we simulated the steady state growth of one quarter of a cell using symmetry conditions appropriate for different array structures, illustrated as cyan dashed boxes in Supplementary Fig. 4. These simulations were performed for a grain oriented along the temperature gradient direction, i.e. with $\theta = \phi = 0°$. For a hexagonal array (Supplementary Fig. 4a), we used a domain size ratio of $L_z/L_y = \sqrt{3}/2$, with $L_y = \lambda/2$ [10,28]. For an FCC-like array (Supplementary Fig. 4b), we used a domain size ratio $L_z/L_y = 1/2$, with $L_y = \lambda\sqrt{2}$. These simulations were performed with no-flux boundary conditions along the y boundaries and the $z = 0$ boundary and anti-symmetry condition along the $z = L_z$ boundary (see Refs. [9,10]). For a thin-sample geometry (Supplementary Fig. 4c) we used $L_y = \lambda/2$ and a fixed $L_z = H/2$ and no-flux boundary conditions along all boundaries except for the sample walls (thick black lines), where we set a simplified geometrical wetting condition by imposing a non-normal angle between the solid-liquid interface and the wall

(see Refs. [10,19,22]):

$$\left.\frac{\partial\psi}{\partial z}\right|_{z=0} = -\left.\frac{\partial\psi}{\partial z}\right|_{z=H} = +1. \tag{25}$$

Then, after obtaining one quarter of a steady state cell for one given λ, we used the resulting fields as initial conditions of a new simulation for a slightly different λ. In order to reduce (increase) λ while keeping numerical parameters Δx and W unchanged for the sake of a consistent numerical accuracy, we restarted simulations with fewer (additional) numerical grid points. Hence, each new simulation was started from ψ and U fields that were either stretched or squeezed and thus bilinearly interpolated from the previous simulation for a grid with a slightly different number of points in y and z dimensions, while the x dimension remained unchanged throughout. We increased L_y from 70 µm to 160 µm for a hexagonal array, and from 70 µm to 211 µm for an FCC array, both with steps of 13 µm. For the thin-sample array, we increased L_y from 67 µm to 163 µm with steps of 6 µm, for a given sample thickness $H = 198$ µm. Finally, we duplicated fields obtained for a steady-state quarter of cell in order to build entire stable arrays, illustrated as green thin boxes in Supplementary Fig. 4. Then, we imposed crystal angles (α, γ), or (θ, ϕ), in order to investigate the drift dynamics of cells in an entire array. Periodic boundary conditions were imposed along the y and z axes for hexagonal and FCC arrays. The simulations of the thin-sample array has periodic conditions along the y boundaries and an imposed wetting angle along the sample walls at the z boundaries, following Eq. (25). We use these simulations to study the selection of the cell growth direction, i.e. the pattern drift direction and velocity linked to θ_d, as discussed later.

Initial conditions for a spatially-extended bi-crystal. To perform bi-crystal simulations such as in Fig. 3 and Fig. 4 of the Letter, we used a similar method as described in Ref. [19]. The boundary conditions were symmetric (i.e. no-flux) for the x and y boundaries and periodic for the z boundaries. First, we run a preliminary simulation with both the left (blue) and right (red) grains well-oriented, i.e. with $\theta = \phi = 0°$. A planar two-sided no-flux inner boundary, normal to the y axis, is located between the two grains at $y = y_b$ in order to prevent premature invasion of grains during transient microstructure growth after the destabilization of the planar front [19]. The inner boundary is fixed within the sample frame was set with a length of 2000 µm in the x direction.

The simulation was then performed slightly longer after the inner boundary disappearance in order to allow the pattern to stabilize and avoid transient conditions relative to the removal of this inner wall. At the end of the simulation, i.e. at $t = 3000$ s, the GB was hence relatively straight and normal to the y axis and located around the inner boundary position y_b. For the simulation reproducing experimentally measured grain orientations in Fig. 3 of the Letter, the domain size is $L_x \times L_y \times L_z = 2295 \times 1912 \times 633$ µm^3, and the initial GB is set at $y_b = 478$ µm. For the mapping of GB stability as a function of bi-crystal configurations (ϕ_1, ϕ_2) in Fig. 4b of the main text, the domain size is with $L_y = 1272$ µm with similar L_x and L_z as previously. The initial GB location was then set to $y_b = 636$ µm, i.e. in the middle of L_y. While all simulations presented in Fig. 4b of the Letter were performed with $y_b = L_y/2$, additional simulations discussed later in this document (Supplementary Fig. 6a-c) were performed with $y_b = 3L_y/4 = 959$ µm. We used the resulting ψ, U, and p fields as initial conditions of the bi-crystal simulations in the Letter, only changing the grain orientations to the desired values, i.e. $(\theta_1, \phi_1) = (6°, -56°)$ and $(\theta_2, \phi_2) = (3°, 84°)$ in Fig. 3, and $\theta_1 = \theta_2 = 5°$ with various (ϕ_1, ϕ_2) combinations in Fig. 4b.

Solitary cell in a host hexagonal grain. In order to produce a SC within a foreign hexagonal array, we first built an extended 24-cell array, hence four times larger than the green-thick box in Supplementary Fig. 4**a**, using a similar procedure as described in the previous subsection. From the resulting ψ field obtained for a single grain with $\theta = \phi = 0°$, we used a separate bespoke C++ code to read the ψ field and isolate a single cell, in order to build a grain index field with a single (blue) cell with $p = +1$ and the remaining 23 (red) cells with $p = -1$. Using these fields, we let the new initially imposed index field relax to a steady state by performing a simulation over $t = 3600$ s, thus obtaining a steady-state array structure with a single SC, still with $\theta = \phi = 0°$ for both grains (images in the first row of Supplementary Fig. 8). From there, we restarted the simulation changing the orientation of the blue cell to $\theta_1 = 5°$ and different ϕ_1 while keeping $\theta_2 = \phi_2 = 0°$ in the host (red) array.

Supplementary Notes

Supplementary Note 1: Growth orientation selection in 3D arrays. From the results of PF simulations of a single tilted grain, we show that the scaling laws for the dependence of the growth angle θ_d upon the crystal angle θ derived from confined thin sample experiments [20,21] and validated with similarly confined phase-field simulations [16,18,22,35], still hold for three-dimensional spatially-extended patterns [7]. The scaling law relating the ratio θ_d/θ to the array Péclet number Pe $= \lambda V/D$ writes

$$\frac{\theta_d}{\theta} = 1 - \frac{1}{1 + f\,\mathrm{Pe}^g}, \tag{26}$$

where here we consider f and g as constants. We considered the case of a hexagonal array (Supplementary Fig. 5a), a FCC-like array (Supplementary Fig. 5b) and a confined thin-sample array (Supplementary Fig. 5c). Red and blue arrows in Supplementary Fig. 5a-c illustrate drift directions ϕ that were considered. In Supplementary Figs. 5d-f, we plot θ_d/θ as a function of Pe for different θ using a hexagonal array (d), for different array symmetry with $\theta = 10°$ (e), and for different drift angles ϕ in hexagonal and FCC arrays (f). All data points in Supplementary Figs. 5d-f collapse onto one master curve defined by Eq. (26) and shown as the black solid line, with values of $f = 0.67$ and $g = 1.47$ obtained from the least-square fit of the PF results with a hexagonal array for (θ, ϕ) = (10°, 0°). These results confirm that Eq. (26) still stands for 3D configurations, and hence provide a direct way to correlate the crystal orientation angle θ input to our PF simulations with the growth direction angle θ_d estimated from drifting velocities measured in the experiments.

Supplementary Note 2: Grain boundary stability. *Influence of* θ. First, we investigated the influence of θ on the GB stability. Restarting from a simulation with a straight GB located between two well-oriented grains, we imposed different crystal orientations $\theta_2 > 0°$ with $\phi_2 = 0°$ for one grain while keeping $\theta_1 = \phi_1 = 0°$ for the other grain. Supplementary Fig. 6 shows the resulting microstructures after 3 h for $\theta_2 = 5$, 10, 15, and 20°, as compared to the initial GB location (white dashed line). When $\theta_2 \leq 10°$, red cells penetrate into the blue well-oriented grain (Supplementary Fig. 6a-b). This leads to the morphological destabilization of the initially straight GB (white dashed lines). On the other hand, when $\theta_2 \geq 15°$, the GB remains relatively straight and close to its initial

position (Supplementary Fig. 6c-d). This is consistent with the results of previous 2D and confined-3D thin sample simulations [19], which showed that a converging GB has a high mobility only in the vicinity of a symmetrical configuration, e.g. for $\theta_1 + \theta_2 = 0°$ and $\phi_1 = \phi_2 = 0°$. In the case of Supplementary Fig. 6, since $\theta_1 = 0°$, the converging GB only exhibits a high mobility in the vicinity of $\theta_2 \approx 0°$, namely here for $\theta_2 \leq 10°$. This is consistent with the experiment in Fig. 1c of the Letter, in which the left and right grains respectively have $\theta = 6$ and 3°. It is also worth noting that the penetration of a grain is expected to depend upon the primary spacing of the arrays [17,36], which is $\lambda = 189$ µm in these simulations.

Influence of ϕ. As explained above, with the current parameters the (inter-)penetration of grain can only be observed for $\theta_2 \leq 10°$. Hence, we studied the influence of ϕ_1 and ϕ_2 in bi-crystalline simulations with $\theta_1 = \theta_2 = 5°$ (Fig. 4b of the Letter). Results of the simulations with a GB initially located near the middle of the simulation domain L_y (white dashed line) after 3 h of growth are illustrated in Supplementary Fig. 7. The orientation of the blue grain is mapped from $\phi_1 = 0°$ (left column) to $75°$ (right column). The orientation of the red grain is mapped from $\phi_2 = -90°$ (bottom row) to $+90°$ (top row). These configurations are sufficient to reconstruct the entire (ϕ_1, ϕ_2) space by symmetry and produce Fig. 4b of the Letter. When one grain has $\phi \approx 0°$, i.e. when its drift direction is directly pointing towards the neighbor grain, groups of cells within the $\phi \approx 0°$ grain systematically penetrate the neighbor grain. This can be seen for instance with $\phi_1 = 0°$ in the leftmost column of Supplementary Fig. 7, where the left (blue) grain systematically invades the right (red) grain. Similarly, within the same first column, the right (red) grain may also penetrate the left (blue) grain, but only when its ϕ_2 angle is also low enough, i.e. here for $\phi_2 \leq 60°$. On the other hand, when $\phi_2 \geq 75°$ for either of the grains, the penetration process remains limited. Subsequently, the boundaries that remain the most stable and straight are for cases with both high ϕ_1 and high ϕ_2, i.e. in the top-right and bottom-right corners of Supplementary Fig. 7. For cases with $\phi_2 = \pm 90°$ and $\phi_1 = 90°$, which are not shown in Supplementary Fig. 7, the GB remains stable and straight.

Supplementary Note 3: Solitary cells. *SC emergence.* In Supplementary Fig. 7, yellow circles identify the emergence of a SC or that of an isolated group of cells drifting inside the neighbor grain. The observed region for possible emergence of a SC is represented with the thick green line, namely (1) for $\phi_1 + \phi_2 < 90°$ and (2) for both $|\phi_1| < 75°$ and $|\phi_2| < 75°$.

Solitary cell behavior. We explored the behavior of an isolated SC as a function of the host array spacing λ and of the SC drift direction ϕ_1. In order to investigate these SC behaviors, we considered a SC within the host hexagonal array. The initial arrays had spacings λ = 122, 128, 134, 141, 147, 160, 192, 224, and 256 µm, and cells in the host grain were favorably oriented (i.e. $\phi_2 = \theta_2 = 0°$). Then, we varied drift directions of one SC from $\phi_1 = 0°$ (close-packed direction) to $30°$ at a fixed $\theta_1 = 5°$. These simulations were carried out for at least 3 h of growth — some of them for up to 9 h when necessary to unambiguously classify the behavior of the SC. Supplementary Fig. 8 illustrates three different behaviors for four different configurations (columns), starting from $t = 0$ h (top row) to later times (bottom rows). The initial array spacing is fixed at $\lambda = 141$ µm for Supplementary Fig. 8a-c. These results illustrate that the SC behavior depends upon its drift direction ϕ_1 (white arrows in Supplementary Fig. 8). For a higher $\phi_1 = 30°$ (a), a SC drifts laterally until it is eliminated at 4.4 h. Otherwise, a SC for a low $\phi_1 = 0°$ (b) and $15°$ (c) may progress as it eliminates neighbor cells (cross symbols). Even if a SC has a higher angle $\phi_1 = 30°$, it may survive indefinitely when the host array has a larger spacing, e.g. for $\lambda = 192$ µm (d). In Supplementary Figs. 8b-c, one host grain cell to be eliminated is identified with cross symbols on the center row. This elimination may either affect a directly adjacent host cell if the drifting direction of the SC is closely directed toward the host cell, e.g. for $\phi_1 = 0°$ (b), or the SC may first squeeze its way between initially adjacent cells before eliminating the host cell with the location closest to the SC path, e.g. for $\phi_1 = 15°$ (c). The resulting (ϕ_1, λ) map in Supplementary Fig. 9 shows three distinct behaviors. If an array has a spacing lower than the low spacing stability limit λ_{min}, a cell in the array is eliminated and the array spacing becomes higher [3,15,27,37]. We independently performed simulations to measure the limit of a hexagonal array, which yields $\lambda_{min} = 112$ µm (gray dashed line). For SC simulations, when the initial spacing is closest to λ_{min} (gray region), the SC is systematically eliminated as it tries to drift laterally within the array. On

the other hand, when λ is high enough (green region), the SC can move within the array and progresses as an accommodating SC by squeezing itself between red cells, without any elimination event. We expect that the SC would remain as an accommodating SC up to the higher spacing limit $\lambda_{max} = 342$ µm (black dashed line). An array with $\lambda > \lambda_{max}$ would thus exhibit a sidebranching instability [3,15,27,37]. Within a narrow range of intermediate spacings (orange region), the SC progresses as a dominant SC by eliminating its first neighbor within the host array. The dominant SC regime is the most common in experiments, while in simulations the SC often proceeds as an accommodating SC. We attribute this discrepancy between experiments and simulations to potential uncertainties in material properties and thermal conditions, which are not directly measured during the experiments. For computational efficiency, present calculations consider a frozen temperature approximation (i.e. a one-dimensional temperature field with constant G and V) and do not include the effect of latent heat release during solidification. Yet, previous studies have shown that relaxing these approximations may lead to a modest increase of the dynamically selected primary spacing [3,12]. The conclusions drawn here regarding a SC behavior are valid for the specific case of a perfect hexagonal array and a perfectly oriented host grain. It is expected that, as the topology of the array becomes more complex, for instance due to successive elimination and/or branching events, or when the host grain is also tilted, the classification of the SC behavior becomes more complex too.

Supplementary Figures

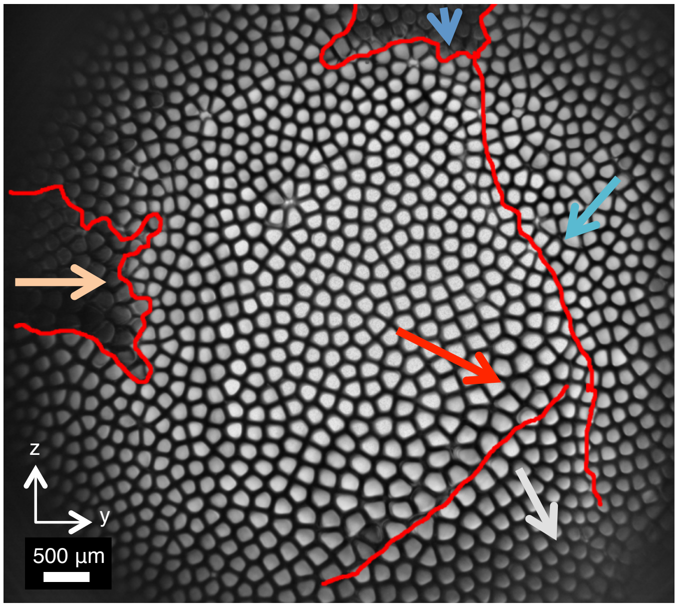

Supplementary Figure 1 Solid-liquid interface pattern of a SCN-0.24wt% camphor alloy at $V = 2.0$ µm/s and $G = 19$ K/cm. Solid-liquid interface seen from the liquid, facing the main growth direction. A group of cells (hereafter called grain) drifts in a same direction (arrows) due to their crystal orientation, and lateral dynamics of these groups lead to the evolution of boundaries (red lines) between them.

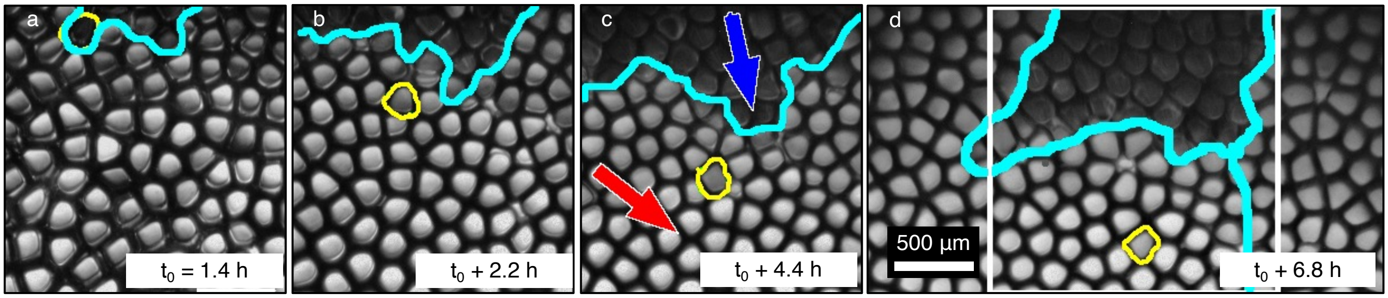

Supplementary Figure 2 Dynamics of a solitary cell (SC) at $V = 2.0$ µm/s and $G = 19$ K/cm. As also observed in Fig. 2a of the Letter for $V = 1.5$ µm/s, a SC emerges in the experiment at $V = 2.0$ µm/s. While the upper grain invades the lower grain (left to right), a cell (circled in yellow) near the convergent GB (cyan line) detaches from its original grain and become a SC (a). This SC survives and drifts within the lower host grain (b-d) until the end of the experiment. Arrows on top of c indicate the overall drift direction of these grains. The white box in d represents the region of a-c at earlier times.

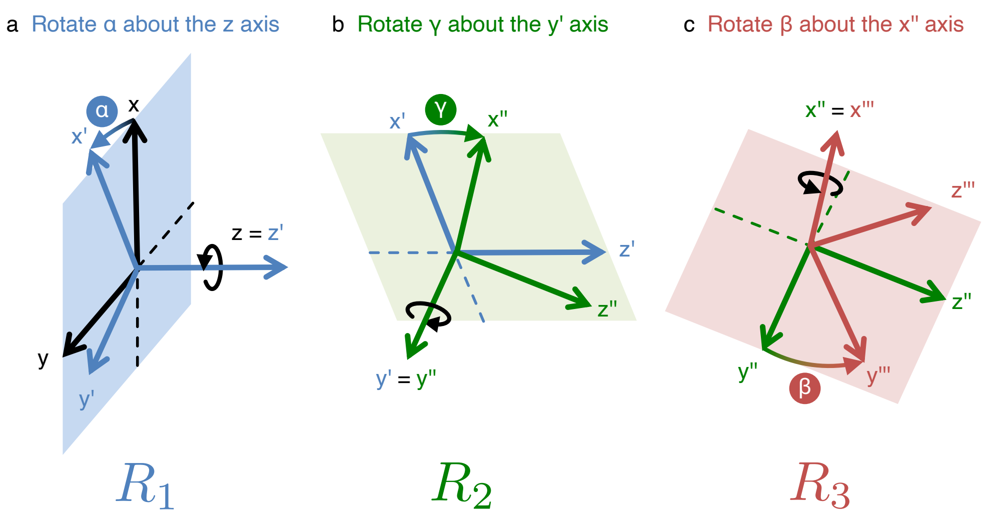

Supplementary Figure 3 **Rotated crystal axes (x''', y''', z''') with respect to the fixed Cartesian axes (x, y, z).** The crystal orientation axes (x''', y''', z''') are obtained by successively applying rotations: R_1 by an angle α about the z axis, resulting in axes (x', y', z') illustrated in a; R_2 by an angle γ about the y' axis, resulting in axes (x'', y'', z'') illustrated in b; R_3 by an angle β about the x'' axis, resulting in axes (x''', y''', z''') illustrated in c. Therefore, the red arrows in c indicate the rotated crystal axes.

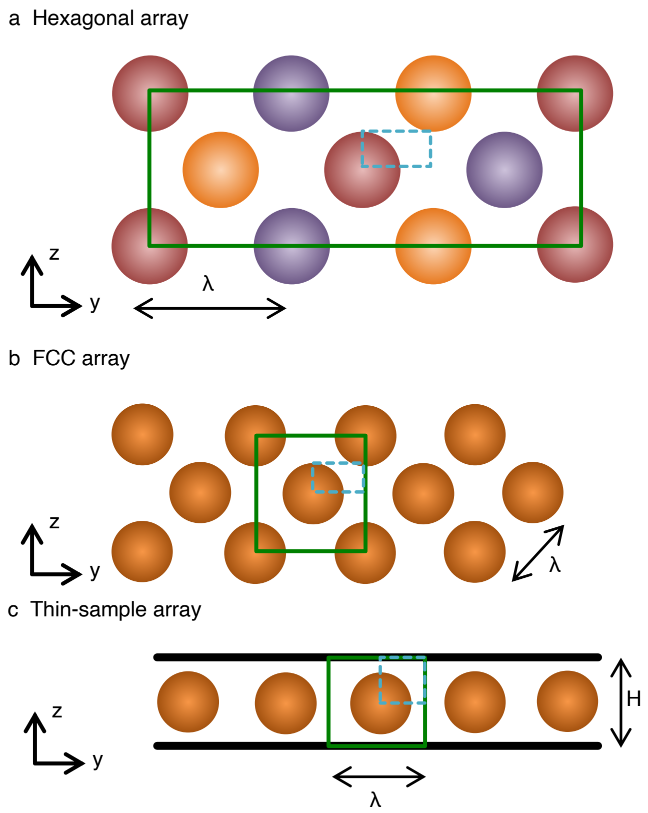

Supplementary Figure 4 **Schematics of different 3D pattern symmetries.** We considered three symmetric patterns of hexagonal (a), FCC-like (face centered cubic) (b), and thin-sample (c) arrays. The thicker black lines in c indicate the sample walls. We performed PF simulations with a domain (green square and cyan square) using symmetry conditions (details in Supplementary Methods).

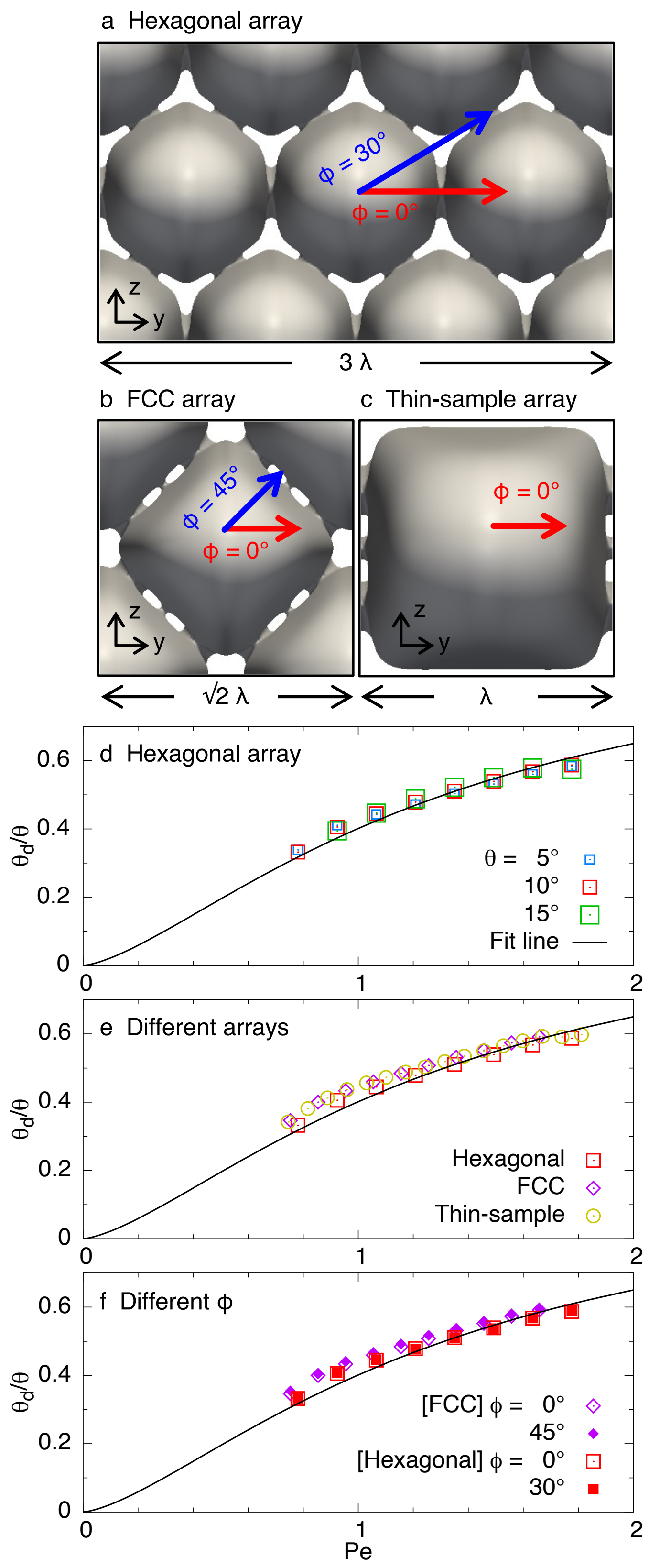

a Hexagonal array
ϕ = 30°
ϕ = 0°
z
y
3 λ
b FCC array
ϕ = 45°
ϕ = 0°
z
y
√2 λ
c Thin-sample array
ϕ = 0°
z
y
λ
d Hexagonal array
θ = 5°
10°
15°
Fit line
θd/θ
0.6
0.4
0.2
0
0
1
2
e Different arrays
Hexagonal
FCC
Thin-sample
θd/θ
f Different ϕ
[FCC] ϕ = 0°
45°
[Hexagonal] ϕ = 0°
30°
θd/θ
Pe

Supplementary Figure 5 **Lateral drift dynamics of different patterns.** Typical microstructures for different imposed array symmetries, namely hexagonal (a), FCC-like (b), and confined thin sample (c). Drift directions θ_d relative to the crystal orientation θ as a function of the Péclet number Pe $= \lambda V/D$ for different θ (d), array symmetries (e), and projected drifting directions ϕ (f). Thin-sample configurations are with a fixed sample thickness $H = 198$ µm. Simulations in d-e are with $\phi = 0°$ (open symbols). Simulations in e-f are with $\theta = 10°$. Two simulations in f are with $\phi = 30°$ for a hexagonal array and $\phi = 45°$ for a FCC array (closed symbols). The black line in d-f shows the best fit of PF results with a hexagonal array for (θ, ϕ) = (10°, 0°) (red open squares) to Eq. (26), i.e. with $f = 0.67$ and $g = 1.47$.

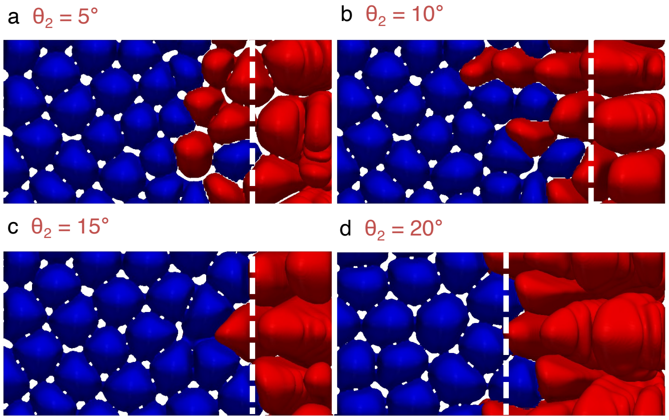

Supplementary Figure 6 GB morphology and stability with respect to the lateral drift of a grain. Images show the microstructures after 3 h of growth using $\theta_2 = 5°$ (a), $10°$ (b), $15°$ (c), and $20°$ (d) for the right (red) grain. In all cases $\theta_1 = \phi_1 = \phi_2 = 0°$. White dashed lines indicate the initial GB position.

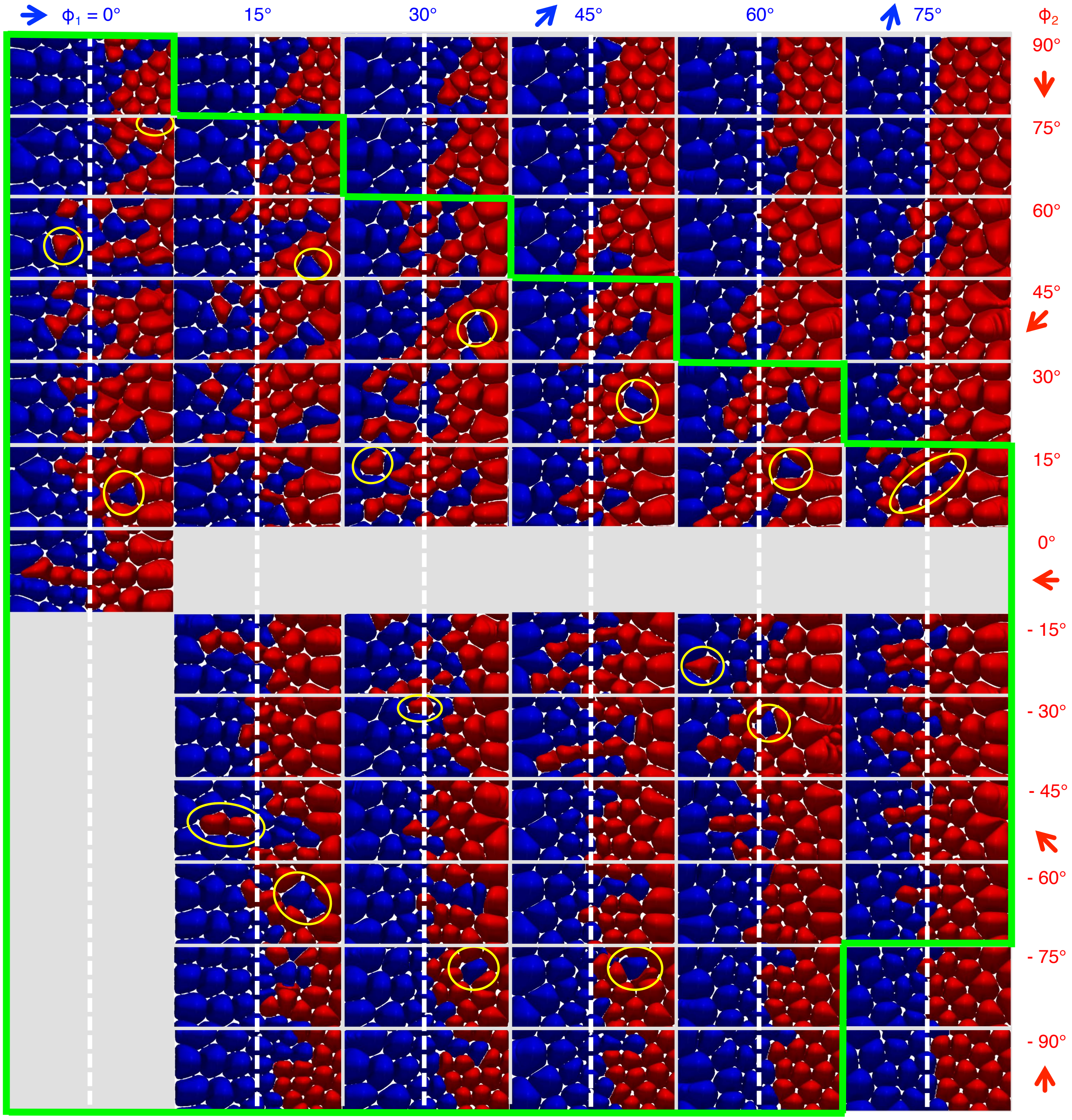

φ1 = 0°
15°
30°
45°
60°
75°
φ2
90°
75°
60°
45°
30°
15°
0°
- 15°
- 30°
- 45°
- 60°
- 75°
- 90°

Supplementary Figure 7 Bi-crystalline microstructures for different pattern drift angles (ϕ_1, ϕ_2). Images show simulated bi-crystalline microstructures after 3 h of growth as a function of crystal orientation angles ϕ_1 for the blue grain and ϕ_2 for the red grain. The GB is initially located at the center of the domain (white dashed lines). All simulations are with $\theta_1 = \theta_2 = 5°$. Solitary cells or groups are outlined in yellow. The green solid line delimits the region of observed emergence of SCs.

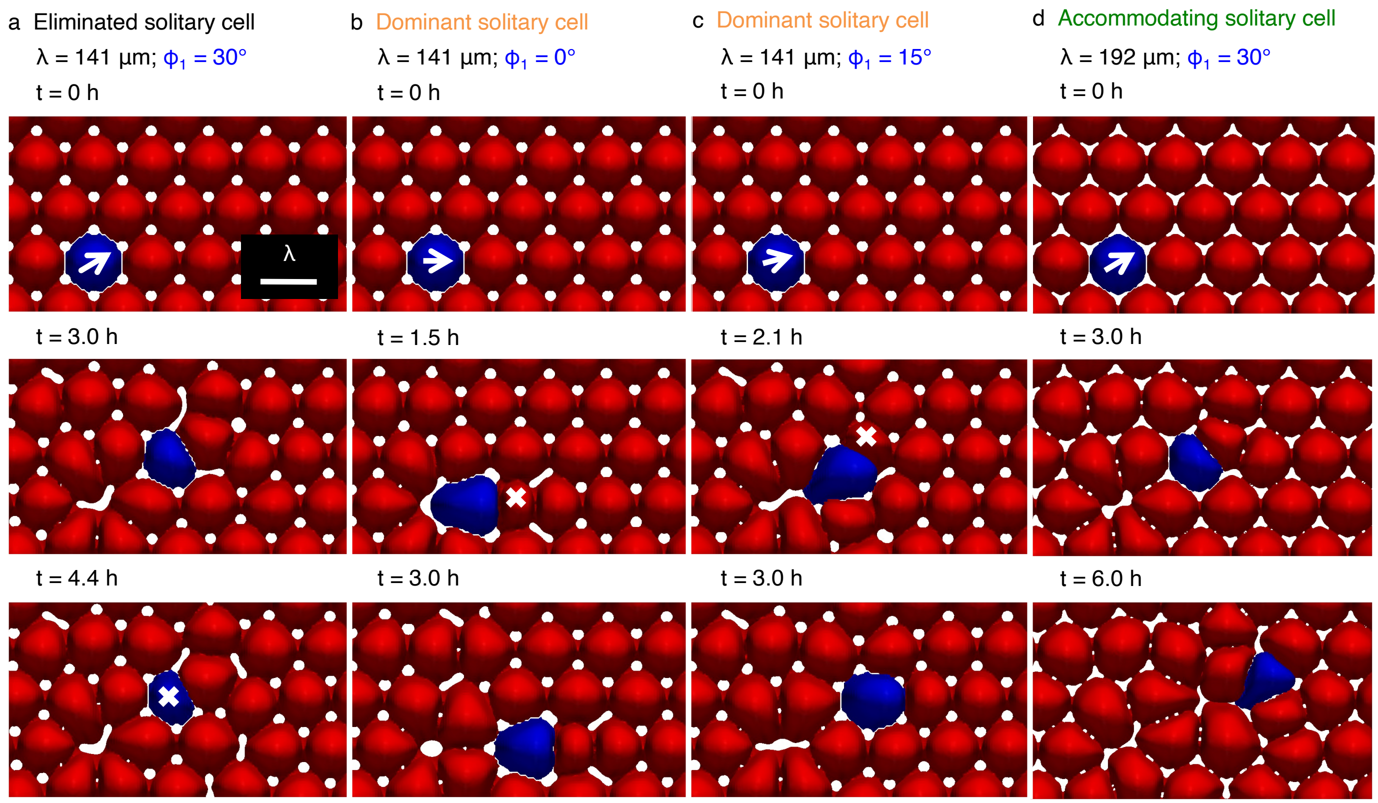

Supplementary Figure 8 **Behaviors of SCs with respect to their drift direction ϕ_1 and to the host grain spacing λ.** Images show simulated evolution of a (blue) SC within a (red) host array as time elapses (top to bottom row). These simulations result in either: the elimination of the SC (a), a dominant SC that eliminates cells within the host array (b-c), or an accommodating SC that squeezes between the host array as it progresses without any elimination event (d). Red hexagonal arrays are with $\lambda = 141$ µm (a-c) and 192 µm (d). Drifting directions of SCs (white arrows on top-row images) are $\phi_1 = 0°$ (b), 15° (c), and 30° (a, d), while $\theta_1 = 5°$ and $\theta_2 = \phi_2 = 0°$. White crosses mark the eliminated cells shortly before their elimination.

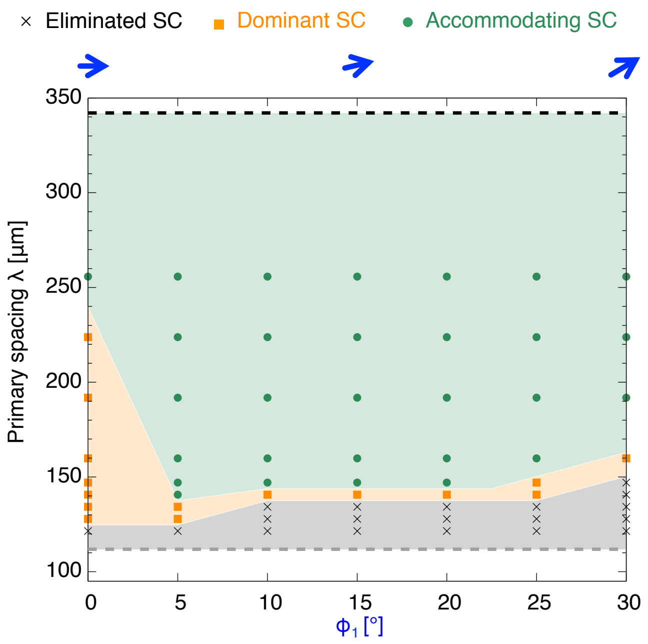

Supplementary Figure 9 **SC behaviors within a hexagonal host array.** Using PF simulations, we identify three distinct behaviors (different colors) of SCs within a well-oriented ($\theta_2 = \phi_2 = 0°$) hexagonal array. SCs are set with different ϕ_1 for a fixed $\theta_1 = 5°$. Blue arrows above the graph illustrate the drift direction ϕ_1. Simulations result in either: SC elimination (thin black crosses), or SC surviving with (orange squares) and without (green circles) elimination of cells in the host matrix. Horizontal gray and black dashed lines respectively mark the minimum λ_{min} and maximum λ_{max} stable spacing limits identified for a perfect hexagonal well-oriented array.

Supplementary Tables

Supplementary Table 1: Material and numerical parameters for a SCN-0.24wt% camphor alloy at $V = 1.5$ μm/s and $G = 19$ K/cm.

Parameter	Symbols	Value	Unit
Liquidus slope	m	-1.365	K/wt%
Diffusivity	D	270	$\mu m^2/s$
Gibbs-Thomson coefficient	Γ	64.78	K μm
Partition coefficient	k	0.07	
Anisotropy strength	ε_4	0.011	
Interface thickness	W	179	d_0
Grid spacing	Δx	1.2	W
		3.2	μm
Time step	Δt	0.00567	s
Noise strength	F_ψ	0.01	

Supplementary Table 2: Crystal orientations used in PF simulations. We imposed angles α and γ into the PF model. These values are converted to spherical angles θ and ϕ (Supplementary Figs. 3 and Fig. 4a of the Letter).

α [°]	γ [°]	θ [°]	ϕ [°]
0.0	0.0	0.0	0.0
5.0	0.0	5.0	0.0
4.9	0.4	4.9	4.7
4.9	0.9	5.0	10.4
4.9	1.3	5.1	14.9
4.7	1.7	5.0	19.9
4.5	2.1	5.0	25.0
4.5	2.6	5.2	30.1
3.6	3.6	5.1	45.1
2.6	4.5	5.2	60.0
1.3	4.9	5.1	75.2
0.0	5.0	5.0	90.0
10.0	0.0	10.0	0.0
8.7	5.0	10.0	30.0
7.1	7.1	10.0	45.2
15.0	0.0	15.0	0.0
0.3	2.8	2.8	83.9
3.5	5.1	6.2	55.6

Supplementary References

1. Mullins, W. W. & Sekerka, R. F. Stability of a planar interface during solidification of a dilute binary alloy. *Journal of Applied Physics* **35**, 444–451 (1964). URL https://doi.org/10.1063/1.1713333. https://doi.org/10.1063/1.1713333.

2. Tiller, W. A., Jackson, K. A., Rutter, J. W. & Chalmers, B. The redistribution of solute atoms during the solidification of metals. *Acta Metallurgica* **1**, 428–437 (1953). URL http://dx.doi.org/10.1016/0001-6160(53)90126-6.

3. Song, Y. *et al.* Thermal-field effects on interface dynamics and microstructure selection during alloy directional solidification. *Acta Materialia* **150**, 139–152 (2018). URL https://doi.org/10.1016/j.actamat.2018.03.012.

4. Bergeon, N. *et al.* Dynamics of interface pattern formation in 3D alloy solidification: first results from experiments in the DECLIC directional solidification insert on the international space station. *Journal of Materials Science* **46**, 6191–6202 (2011). URL http://dx.doi.org/10.1007/s10853-011-5382-2.

5. Bergeon, N. *et al.* Dynamical microstructure formation in 3D directional solidification of transparent model alloys: in situ characterization in DECLIC directional solidification insert under diffusion transport in microgravity. *IOP Conference Series: Materials Science and Engineering* **84**, 012077 (2015). URL https://dx.doi.org/10.1088/1757-899X/84/1/012077.

6. Bottin-Rousseau, S., Akamatsu, S. & Faivre, G. Dynamical polygonization below the cellular-bifurcation threshold in thin-sample directional solidification. *Physical Review B* **66**, 054102 (2002). URL https://link.aps.org/doi/10.1103/PhysRevB.66.054102.

7. Mota, F. *et al.* Effect of sub-boundaries on primary spacing dynamics during 3D directional solidification conducted on DECLIC-DSI. *Acta Materialia* **204**, 116500 (2021). URL https://doi.org/10.1016/j.actamat.2020.116500.

8. Faivre, G., Bottin-Rousseau, S. & Akamatsu, S. The trajectory of subboundary grooves during directional solidification of dilute alloys. *Comptes Rendus Physique* **14**, 149–155 (2013). URL https://doi.org/10.1016/j.crhy.2013.01.003.

9. Bergeon, N. *et al.* Spatiotemporal dynamics of oscillatory cellular patterns in three-dimensional directional solidification. *Physical Review Letters* **110**, 226102 (2013). URL https://link.aps.org/doi/10.1103/PhysRevLett.110.226102.

10. Tourret, D. *et al.* Oscillatory cellular patterns in three-dimensional directional solidification. *Physical Review E* **92**, 042401 (2015). URL https://link.aps.org/doi/10.1103/PhysRevE.92.042401.

11. Pereda, J. *et al.* Experimental observation of oscillatory cellular patterns in three-dimensional directional solidification. *Physical Review E* **95**, 012803 (2017). URL https://link.aps.org/doi/10.1103/PhysRevE.95.012803.

12. Mota, F. L. *et al.* Initial transient behavior in directional solidification of a bulk transparent model alloy in a cylinder. *Acta Materialia* **85**, 362–377 (2015). URL https://doi.org/10.1016/j.actamat.2014.11.024.

13. Mota, F. *et al.* Quantitative determination of the solidus line in the dilute limit of succinonitrile–camphor alloys. *Journal of Crystal Growth* **447**, 31–35 (2016). URL https://doi.org/10.1016/j.jcrysgro.2016.04.054.

14. Lu, S.-Z. & Hunt, J. D. A numerical analysis of dendritic and cellular array growth: the spacing adjustment mechanisms. *Journal of Crystal Growth* **123**, 17–34 (1992). URL http://dx.doi.org/10.1016/0022-0248(92)90006-5.

15. Hunt, J. D. & Lu, S. Z. Numerical modeling of cellular/dendritic array growth: spacing and structure predictions. *Metallurgical and Materials Transactions A* **27**, 611–623 (1996). URL http://dx.doi.org/10.1007/BF02648950.

16. Tourret, D. & Karma, A. Growth competition of columnar dendritic grains: A phase-field study. *Acta Materialia* **82**, 64–83 (2015). URL https://doi.org/10.1016/j.actamat.2014.08.049.

17. Takaki, T. *et al.* Large-scale phase-field studies of three-dimensional dendrite competitive growth at the converging grain boundary during directional solidification of a bicrystal binary alloy. *ISIJ International* **56**, 1427–1435 (2016). URL https://doi.org/10.2355/isijinternational.ISIJINT-2016-156.

18. Li, J., Wang, Z., Wang, Y. & Wang, J. Phase-field study of competitive dendritic growth of converging grains during directional solidification. *Acta Materialia* **60**, 1478–1493 (2012). URL http://dx.doi.org/10.1016/j.actamat.2011.11.037.

19. Tourret, D., Song, Y., Clarke, A. & Karma, A. Grain growth competition during thin-sample directional solidification of dendritic microstructures: A phase-field study. *Acta Materialia* **122**, 220–235 (2017). URL https://doi.org/10.1016/j.actamat.2016.09.055.

20. Akamatsu, S. & Ihle, T. Similarity law for the tilt angle of dendrites in directional solidification of non-axially-oriented crystals. *Physical Review E* **56**, 4479–4485 (1997). URL https://link.aps.org/doi/10.1103/PhysRevE.56.4479.

21. Deschamps, J., Georgelin, M. & Pocheau, A. Growth directions of microstructures in directional solidification of crystalline materials. *Physical Review E* **78**, 011605 (2008). URL https://link.aps.org/doi/10.1103/PhysRevE.78.011605.

22. Ghmadh, J. *et al.* Directional solidification of inclined structures in thin samples. *Acta Materialia* **74**, 255–267 (2014). URL https://doi.org/10.1016/j.actamat.2014.04.023.

23. Karma, A. & Rappel, W.-J. Phase-field method for computationally efficient modeling of solidification with arbitrary interface kinetics. *Physical Review E* **53**, R3017–R3020 (1996). URL https://link.aps.org/doi/10.1103/PhysRevE.53.R3017.

24. Karma, A. & Rappel, W.-J. Quantitative phase-field modeling of dendritic growth in two and three dimensions. *Physical Review E* **57**, 4323–4349 (1998). URL https://link.aps.org/doi/10.1103/PhysRevE.57.4323.

25. Karma, A. Phase-field formulation for quantitative modeling of alloy solidification. *Physical Review Letters* **87**, 115701 (2001). URL https://link.aps.org/doi/10.1103/PhysRevLett.87.115701.

26. Echebarria, B., Folch, R., Karma, A. & Plapp, M. Quantitative phase-field model of alloy solidification. *Physical Review E* **70**, 061604 (2004). URL https://link.aps.org/doi/10.1103/PhysRevE.70.061604.

27. Echebarria, B., Karma, A. & Gurevich, S. Onset of sidebranching in directional solidification. *Physics Review E* **81**, 021608 (2010). URL https://link.aps.org/doi/10.1103/PhysRevE.81.021608.

28. Gurevich, S., Karma, A., Plapp, M. & Trivedi, R. Phase-field study of three-dimensional steady-state growth shapes in directional solidification. *Physical Review E* **81**, 011603 (2010). URL https://link.aps.org/doi/10.1103/PhysRevE.81.011603.

29. Glasner, K. Nonlinear preconditioning for diffuse interfaces. *Journal of Computational Physics* **174**, 695–711 (2001). URL http://dx.doi.org/10.1006/jcph.2001.6933.

30. Nickolls, J., Buck, I., Garland, M. & Skadron, K. *Scalable Parallel Programming with CUDA* (Queue, 2008).

31. Sanders, J. & Kandrot, E. *CUDA by example: an introduction to general-propose GPU programming* (Addison-Wesley Professional, 2010).

32. Shimokawabe, T. *et al.* Peta-scale phase-field simulation for dendritic solidification on the TSUBAME 2.0 supercomputer. In *Proceedings of 2011 International Conference for High Performance Computing, Networking, Storage and Analysis*, SC

'11 (Association for Computing Machinery, New York, NY, USA, 2011). URL https://doi.org/10.1145/2063384.2063388.

33. Karma, A., Lee, Y. H. & Plapp, M. Three-dimensional dendrite-tip morphology at low undercooling. *Physical Review E* **61**, 3996–4006 (2000). URL https://link.aps.org/doi/10.1103/PhysRevE.61.3996.

34. Mota, F. L. *et al.* Convection effects during bulk transparent alloy solidification in declic-dsi and phase-field simulations in diffusive conditions. *JOM* **69**, 1280–1288 (2017). URL http://dx.doi.org/10.1007/s11837-017-2395-6.

35. Song, Y., Akamatsu, S., Bottin-Rousseau, S. & Karma, A. Propagative selection of tilted array patterns in directional solidification. *Physical Review Materials* **2**, 053403 (2018). URL https://link.aps.org/doi/10.1103/PhysRevMaterials.2.053403.

36. Hu, S. *et al.* Competitive converging dendrites growth depended on dendrite spacing distribution of Ni-based bi-crystal superalloys. *Journal of Alloys and Compounds* **735**, 1878–1884 (2018). URL https://doi.org/10.1016/j.jallcom.2017.11.316.

37. Clarke, A. *et al.* Microstructure selection in thin-sample directional solidification of an Al-Cu alloy: In situ x-ray imaging and phase-field simulations. *Acta Materialia* **129**, 203–216 (2017). URL https://doi.org/10.1016/j.actamat.2017.02.047.
