# Supplementary material for: Cell invasion during competitive growth of polycrystalline solidification patterns
Source: Nat Commun. 2023 Apr 19;14:2244. doi: 10.1038/s41467-023-37458-0 (PMC10115863; doi:10.1038/s41467-023-37458-0)
Supplement: Supplementary file 3 — Description of Additional Supplementary Files [file 41467_2023_37458_MOESM3_ESM.pdf]

Description of Additional Supplementary Files

File Name: Supplementary Movie 1
Description: an experimental video showing in situ observations during microgravity directional solidification of a succinonitrile (SCN)-0.24wt% camphor alloy at a velocity *V* = 1.5 µm/s within a temperature gradient *G* = 19 K/cm, from *t* = 0 to 11 h (Fig. 1 in the Letter). These optical images were recorded from a camera with an immersed lens in the liquid directly facing the solidification front main growth direction.

File Name: Supplementary Movie 2
Description: an experimental video showing the emergence and drifting of a solitary cell (SC), corresponding to Fig. 1d-h in the Letter, from *t* = 3.5 to 11 h (SCN-0.24wt% camphor alloy, *V* = 1.5 µm/s, *G* = 19 K/cm). The SC drifts within the neighbor host grain and ultimately is eliminated by being squeezed between two cells from two different grains.

File Name: Supplementary Movie 3
Description: an experimental video showing the emergence and progression of a solitary cell (SC) during microgravity directional solidification of a SCN-0.24wt% camphor alloy at *V* = 1.5 µm/s and *G* = 19 K/cm within a foreign host grain from *t* = 3.5 to 11 h (Fig. 2a of the Letter).

File Name: Supplementary Movie 4
Description: an experimental video showing the early stages of planar interface destabilization, followed by the emergence and drifting of a SC during directional solidification of a SCN-0.24wt% camphor alloy with *V* = 2.0 µm/s and *G* = 19 K/cm from *t* = 0 to 8.2 h (Supplementary Fig. 1).

File Name: Supplementary Movie 5
Description: shows the threedimensional locations of cell tips post-processed from the experiment of Fig. 2 in the Letter (SCN0.24wt% camphor alloy, V = 1.5 µm/s, G = 19 K/cm, from *t* = 3.5 to 11 h). Blue and red colors correspond to left and right grains, respectively, in Fig. 2a. The video highlights the roughening and branching of the GB, and the subsequent formation of a tubular GB defect.

File Name: Supplementary Movie 6
Description: shows phase-field simulation results of the microstructure evolution at *V* = 1.5 µm/s and *G* = 19 K/cm of a SCN0.24wt% camphor alloy from *t* = 0 h to *t* = 12 h. The horizontal and vertical domain sizes are L_y = 1912 µm and L_z = 633 µm, respectively. This simulation uses crystal angles, (θ1, ϕ1) = (6°, - 56°) for the left (blue) grain and (θ2, ϕ2) = (3°, 84°) for the right (red) grain, similar to those measured in experiments. A SC emerges at *t* = t0 = 2.6 h (Fig. 3a in the Letter) and drifts within the red host grain, with a drifting direction dictated by its original grain orientation independently of the drifting direction of the host grain. Periodic boundary conditions apply along the top and bottom boundaries.

File Name: Supplementary Movie 7
Description: shows the three-dimensional trajectories of cells (in blue and red lines for the left and right grains) and the resulting GB morphology (cyan surface) in the phase-field simulation of Supplementary Movie 6 and Fig. 3 of the Letter (SCN-0.24wt% camphor alloy, V = 1.5 µm/s, G = 19 K/cm, from t = 2.6 to 12 h). It highlights the roughening and branching of the GB, and the subsequent formation of a tubular GB defect.

File Name: Supplementary Movie 8
Description: shows phase-field simulation results of the evolutions of bicrystalline microstructures with different crystal orientations, corresponding to Fig. 4c-f in the Letter, namely GB stability (0 s to 8 s), grain interpenetration (8 s to 16 s), one-side grain penetration (16 s to 25 s), and SC emergence (25 s to 33 s).
